# Prevalence and genotype distribution of HPV infection from Hangzhou of Zhejiang Province pre- and during COVID-19 pandemic

**DOI:** 10.3389/fpubh.2024.1357311

**Published:** 2024-05-30

**Authors:** Jian Wang, Ke Zhao, Jianping Xia, Fang He, Na Chen, Weijuan Wang, Yanxiu Ma, Xiaoming Sun

**Affiliations:** ^1^Department of Clinical Laboratory, First People's Hospital of Linping District, Hangzhou, Zhejiang, China; ^2^Linping Campus, The Second Affiliated Hospital of Zhejiang University School of Medicine, Hangzhou, China; ^3^Department of Immunology and Pathogen Biology, School of Basic Medical Sciences, Hangzhou Normal University, Hangzhou, China

**Keywords:** human papillomavirus, prevalence, genotype, China, COVID-19

## Abstract

Limited data exist on HPV prevalence and genotyping during the COVID-19 pandemic. A total of 130,243 samples from 129, 652 women and 591 men who visited the First People’s Hospital of Linping District between 2016 and 2022 were recruited. HPV genotypes were detected by polymerase chain reaction (PCR) amplification and nucleic acid molecular hybridization. Then the prevalence characteristics of HPV genotypes and trends in HPV infection rates from 2016 to 2022 were analyzed. Results showed that among the study population, the overall prevalence of HPV infection was 15.29%, with 11.25% having single HPV infections and 4.04% having multiple HPV infections, consistent with previous findings. HPV genotypes exhibited similar distribution patterns in both male and female groups, with HPV16, HPV52, HPV58, HPV18, and HPV39 being the most prevalent. Age-related analysis unveiled a bimodal pattern in HPV prevalence, with peaks in infection rates observed in individuals below 20 and those aged 61–65 years. Comparing the pre- and during COVID-19 periods revealed significant disparities in HPV infections, with variations in specific HPV genotypes, including 16, 18, 35, 45, 52, 58, 59, and 68. This study provides valuable insights into the prevalence, distribution, and epidemiological characteristics of HPV infections in a large population. It also highlights the potential impact of the COVID-19 pandemic on HPV trends.

## Introduction

Human papillomaviruses (HPV) are small groups of double-stranded DNA viruses without enveloped icosahedral capsids that cause the most common sexually transmitted infections among women worldwide. Approximately 90% of HPV infections cause no symptoms and resolve spontaneously within 2 years (1). These viruses are categorized into two distinct groups: low-risk HPVs (LR-HPVs), accountable for anogenital and cutaneous warts, and high-risk HPVs (HR-HPVs), linked to anogenital cancers and oropharyngeal cancers (2). The International Agency for Research on Cancer (IARC) has categorized 12 HPV types, including HPV16, HPV18, HPV31, HPV33, HPV35, HPV39, HPV45, HPV51, HPV52, HPV56, HPV58, and HPV59. The prevalence of cervical cancer, for instance, is overwhelmingly linked to HPV, with HPV16 and HPV18 strains alone contributing to 70% of cases (3, 4). Similarly, a considerable portion, ranging from 60 to 90%, of other cancers mentioned earlier are also connected to HPV (5). Notable among these are HPV6 and HPV11, which frequently cause genital warts and laryngeal papillomatosis (5, 6). Risk factors for persistent infection with sexually transmitted HPV types encompass early onset of sexual activity, multiple sexual partners, smoking, and compromised immune function (7).

The global epidemiological landscape of HPV infections and their associated impact demonstrates significant variation, driven by factors such as geographic location, socioeconomic conditions, cultural practices, genetic variability within the viral genome, as well as intrinsic individual attributes like age, gender, anatomical location, and overall health status (8). A meta-analysis has estimated the global HPV prevalence among women of all ages to be 11.7%. Noteworthy is the higher prevalence in Sub-Saharan Africa (24.0%), Eastern Europe (21.4%), and Latin America (16.1%) (9). The reported prevalence of HPV infections among Chinese mainland women stands at 11.0%, resembling the worldwide average (10).

The prevalence of HPV infections has been of considerable interest, especially concerning how it may have been affected by the COVID-19 pandemic. There are several factors, including healthcare access disruption, lockdowns, and social distancing, impact on vaccination programs, and behavioral changes that could have influenced HPV infection rates before and after the pandemic. It’s important to note that the specific impact of COVID-19 on HPV infection rates would likely vary across different regions and populations. Comprehensive and up-to-date epidemiological studies would be necessary to provide accurate insights into these changes. Therefore, a need for updated information pertaining to type-specific HPV prevalence and distribution across general populations has arisen. To address this, we conducted a retrospective hospital-based study in Zhejiang, China, aimed at assessing the overall prevalence and distribution of HPV types. More importantly, we also aim to investigate the changes before and during the COVID-19 pandemic.

## Materials and methods

### Study subjects

In this study, a total of 132,269 HPV tests were collected. For patients with multiple HPV tests, only the results from their first test were included. Any samples with incomplete information, such as missing age or gender data, were excluded from the study. Additionally, we excluded patients who had undergone a hysterectomy or cone biopsy, as well as those with other sexually transmitted infections like HIV-1 or HSV. 130,243 unique HPV tests were analyzed, comprising 129,652 women and 591 men. These tests were conducted at either the Physical Examination Center or the Gynecology Department of the First People’s Hospital of Linping District, covering the period from January 1st, 2016 to December 31, 2022. All participants willingly opted to undergo an HPV test and actively participated in this research. Prior to their involvement, participants or their guardians were provided with comprehensive information about the study and given their informed consent by signing the required documents. This research was granted approval by the Ethics Committees of the First People’s Hospital of Linping District, the ethics approval number was LPYYLL-2022039. The methods employed throughout the study adhered to pertinent guidelines and standard operating procedures.

### Sample collection, HPV detection, and genotyping

The subjects were asked to refrain from sexual activity and avoid washing their genital area for 48 h prior to sample collection. Cervical samples were obtained by using a cervical brush and male urethral secretion swabs were placed in a preservative buffer solution. The samples were stored at 2–8°C for no more than 7 days. HPV DNA PCR amplification and genotyping were performed using the innovative PCR-based One-step Fast Release technology from Sansure Biotech according to the manufacturer’s instructions. It utilizes real-time fluorescent quantitative PCR to target a total of 15 HPV types. Among these, 13 are classified as high-risk types: HPV-16/18/31/33/35/39/45/51/52/56/58/59/68. Additionally, 2 types, HPV-53 and 66, are categorized as possibly high-risk. Reaction conditions were: 50°C for 2 min, denaturation at 94°C for 5 min, denaturation at 94°C for 15 s, annealing at 57°C for 30 s, 45 cycles, and storage at 4°C. The assessment of HPV status is based on the observation of cycle numbers (Ct) at which the fluorescent signal reaches the specified type-specific threshold. A Ct value of ≤39 indicates a positive HPV result, while a Ct value >39 is interpreted as negative. The ABI7500 instrument was used in this study and quality controls were included in all the experiments, including DNA amplification and genotyping, with positive and negative controls included in the PCR assays.

### Data and statistical analysis

Data analysis was performed using SPSS22.0 software (IBM, United States) and detailed calculations were provided in Supplementary Tables. Data were presented as mean ± standard deviation or frequency and percentage for numerical or discrete variables, respectively. The Chi-square test was used to determine significant differences between groups, If the total sample size is >40 and all the expected frequency values are greater than 5, the Pearson-chi-square value is generally used, and if the total sample size is greater than 40, but there are cells with the expected frequency less than 5, the continuous correction chi-square can be preferred. Differences were considered statistically significant at *p < 0.05, **p < 0.01. The heatmap were analyzed and displayed by Heatmapper online tool.^1^ All figures were generated by GraphPad prism version 9.0 and displayed by adobe illustrator version 2021.

### Ethics statement

The studies involving human participants were reviewed and approved by the Ethics Committees of the First People’s Hospital of Linping District Hangzhou City and signed by Qilei Hu with document number LPYYLL-2022039. Written informed consent to participate in this study was provided by the participants.

## Results

### Prevalence of HPV infection in the study population

**Figure 1:**
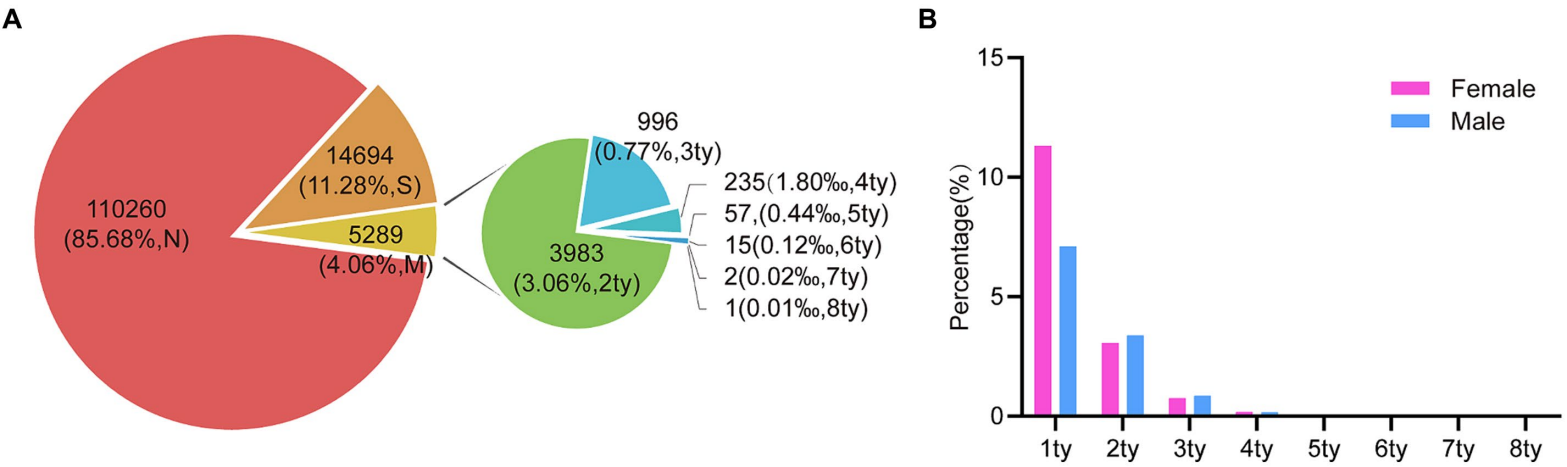
The prevalence of HPV infection in the study population during 2016 and 2022. **(A)** The HPV prevalence among all the specimens (*n* = 130,243). (“N,” HPV Negative; “S,” HPV single infection; “M,” HPV multiple infection; “ty,” HPV genotype). **(B)** The prevalence comparison of HPV single- and multiple-infection in 68 males and 19,915 females.

In this analysis, a total of 130,243 samples were examined. Of these, 129,652 (99.55%) were females, with an average age of 39.39 years (standard deviation: 10.935, range: 8–97), while 591 (0.45%) were males, with an average age of 35.48 years (standard deviation: 9.940, range: 3–84). Among the entire study population, 19,983 (15.34%) patients tested positive for various types of HPV. Among these individuals, 14,694 (11.28%) had single HPV infections, and 5,289 (4.06%) had multiple HPV infections. Within the female subgroup, 19,915 (15.36%) patients tested positive for HPV, with 14,652 (11.30%) having single HPV infections and 5,263 (4.06%) having multiple HPV infections. In contrast, there are 68 (11.51%) patients were found to be HPV positive among the male subgroup, with 42 (7.11%) cases having single HPV infections and 26 (4.40%) cases having multiple HPV infections (Figure 1A; Table 1).

**Table 1:** Baseline characteristics of the study population.

	Total		Female		Male	
	Number (n)	Percentage (%)	Number (n)	Percentage (%)	Number (n)	Percentage (%)
	130,243	100.00	129,652	99.55	591	0.45
Average, years (SD)	39.38 ± 10.938		39.39 ± 10.935		35.48 ± 9.940	
Range	3–97		8–97		3–84	
95% CI for all samples	0.167–0.172		0.168–0.172		0.103–0.158	
HPV positive	19,983	15.34	19,915	15.36	68	11.51
HPV single positive	14,694	11.28	14,652	11.30	42	7.11
HPV multi positive	5,289	4.06	5,263	4.06	26	4.40

We conducted a further classification of patients based on the presence of distinct HPV genotypes. To elaborate, our analysis revealed that among these samples, 3,983 (3.06%) exhibited dual HPV infections, 996 (0.77%) manifested triple HPV infections, 235 (0.18%) with quadruple HPV infections, and 75 (0.058%) displayed infections involving multiple HPV genotypes, with 1 individual even harboring up to eight HPV genotypes (Figure 1A). Notably, this similar trend was observed in both male and female subjects, while individuals harboring multiple HPV genotypes were slightly higher in male groups despite a low number of total positive male individuals (Figure 1B).

### HPV genotype distribution

**Figure 2:**
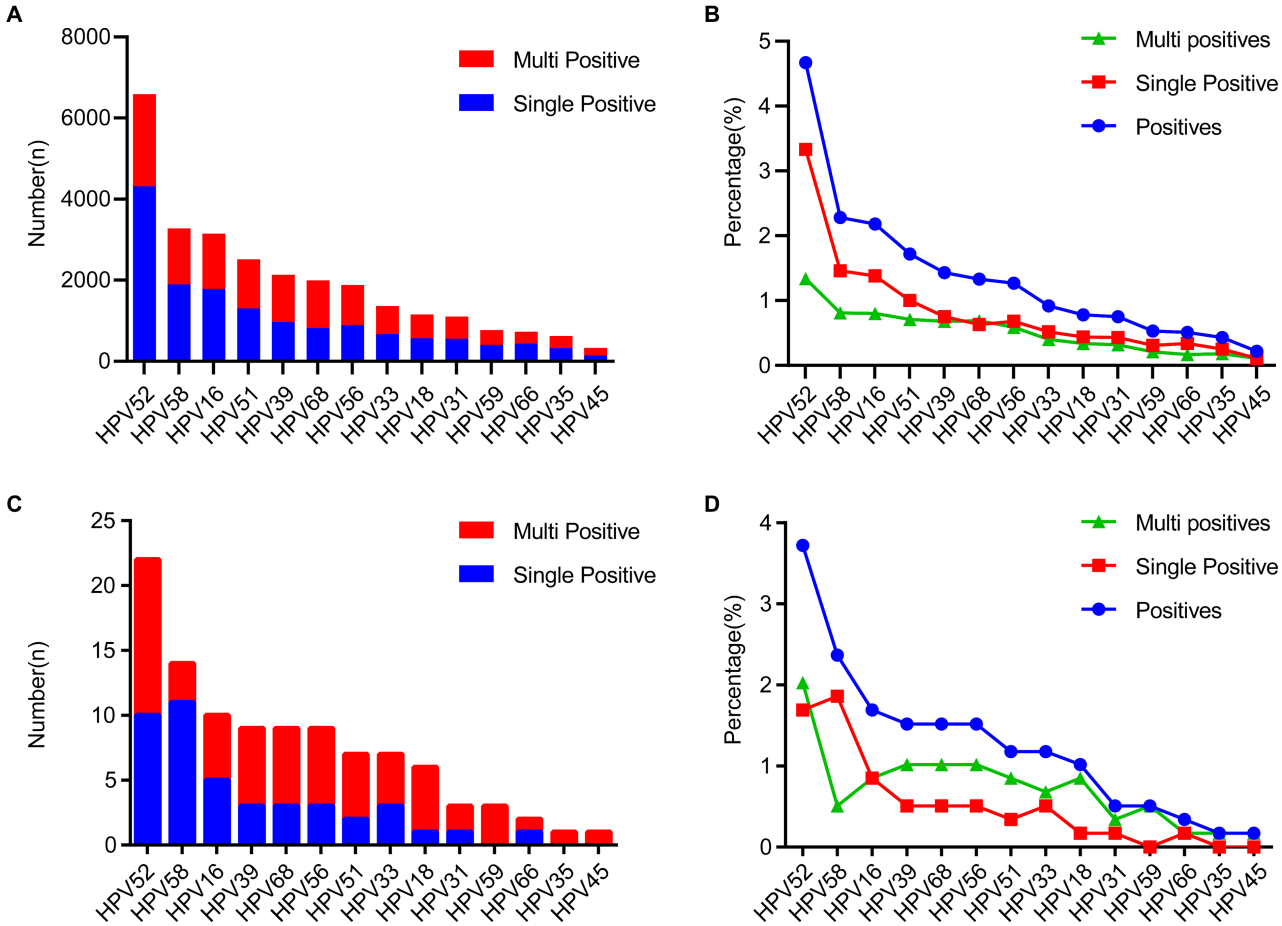
The distribution of HPV genotype among all the HPV-positive specimens. **(A,B)** The prevalence of different HPV genotype and the genotype proportion between single- and multiple-infections in the 19,915 females. **(C,D)** The prevalence of different HPV genotype and the genotype proportion between single- and multiple-infections in the 68 males.

We next investigated the detailed distribution of 14 high-risk genotypes. Notably, we found that the overall distribution of each genotype showed similar trends between the female (Figures 2A,B) and the male group (Figures 2C,D). The top five commonly identified HPV genotypes were consistent in female and male group, including HPV16 (2.18% vs. 1.69%), HPV52 (4.67% vs. 3.72%), HPV58 (2.28% vs. 2.37%), HPV18 (0.78% vs. 1.02%) and HPV39 (1.43% vs. 1.52%). For female group, in the single infection group, HPV52 (3.33%) was the most prevalent subtype, followed by HPV16 (1.38%), HPV58 (1.46%). In the multiple HPV infections group, the most prevalent subtypes were as follows: HPV52 (1.34%), HPV58 (0.81%), HPV16 (0.80%) (Figures 2A,B). In contrast, in the single infection group, HPV58 (1.86%) was the most prevalent subtype, followed by HPV52 (1.69%), HPV16 (0.85%). In the multiple HPV infections group, the most prevalent subtypes were as follows: HPV52 (2.03%), HPV68 (1.02%), HPV39 (1.02%) (Figures 2C,D). To establish whether there exists a gender preference in the infection of distinct HPV genotypes, a stratified chi-square test was conducted. The outcomes revealed no confounding factors between HPV genotypes and gender (Supplementary Table S1). In addition, we also tested whether certain HPV genotypes can be associated with clinical diagnosis in female individuals. We separated clinical diagnosis with irregular menstrual cycles and inflammatory diagnosis, including vaginitis, cervicitis, vulvitis, pelvic inflammatory disease. The results revealed that there are HPV16, HPV58 and HPV59 genotypes associated with irregular menstrual cycle which consistent with previous findings (Supplementary Table S2). Strikingly, HPV16, HPV18, HPV35, HPV45, HPV56, HPV66 and HPV68 showed significant associated with inflammatory diseases (Supplementary Table S3).

### Prevalence of HPV infection in different age groups

**Figure 3:**
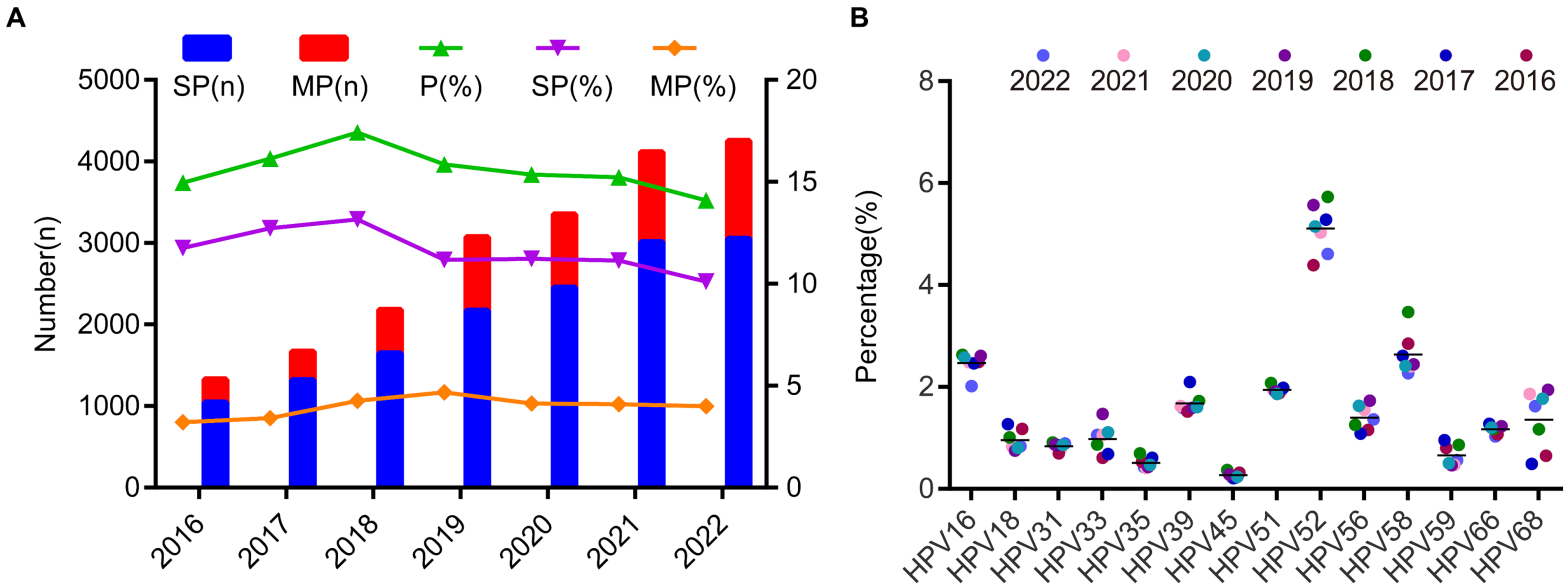
The prevalence of the HPV infection in different age groups. **(A)** The prevalence of HPV single- and multiple-infection in different age groups. **(B)** The heatmap of HPV prevalence rates across different age groups and genotypes. Each row represented deviations of the point prevalence at each age group from its own overall mean. The color reflected the magnitude of the deviation, Some HPV genotype prevalence decreased represented in blue, and others increased, represented in red. The darker the color, the larger difference from its overall mean.

Subsequently, we conducted an in-depth analysis of age-related trends within a dataset comprising 12,043 HPV-positive samples. Our examination of age distribution unveiled a distinctive bimodal pattern in overall HPV prevalence, single infection rates, and multiplicity rates, as visually represented in Figure 3A and Supplementary Table S4. The data unveiled a striking spike in infection rates among individuals below the age of 20 (37.55% for overall prevalence, 20.11% for single positivity, and 17.45% for multiplicity rate). As age increased, infection rates exhibited a consistent decline, reaching a nadir in the 36–40 age group (13.27% for overall prevalence, 10.48% for single positivity, and 2.79% for multiplicity rate). Intriguingly, a subsequent upward trend emerged, culminating in a second peak in the 61–65 age group (21.47% for overall prevalence, 14.30% for single positivity, and 7.17% for multiplicity rate). Notably, the second peak in multiple positive infections was less pronounced. Excluding the <20 age group, which displayed a substantial 17.45% prevalence of multiple positive infections, other age groups-maintained rates below 7.55%.

Furthermore, we also employed heatmap analyses to visualize HPV prevalence rates across different age groups and genotypes. We found that HPV52 is predominant across all age groups, followed by HPV 58 and HPV16. Interestingly, we observed a trend that HPV51 genotype is higher in individuals younger than 55 and then the prevalence decreased after that. In contrast, HPV56 showed the opposite trends (Figure 3B; Supplementary Table S4).

### HPV infection rate from 2016 to 2022 and HPV epidemiological features pre- and during COVID-19

**Figure 4:**
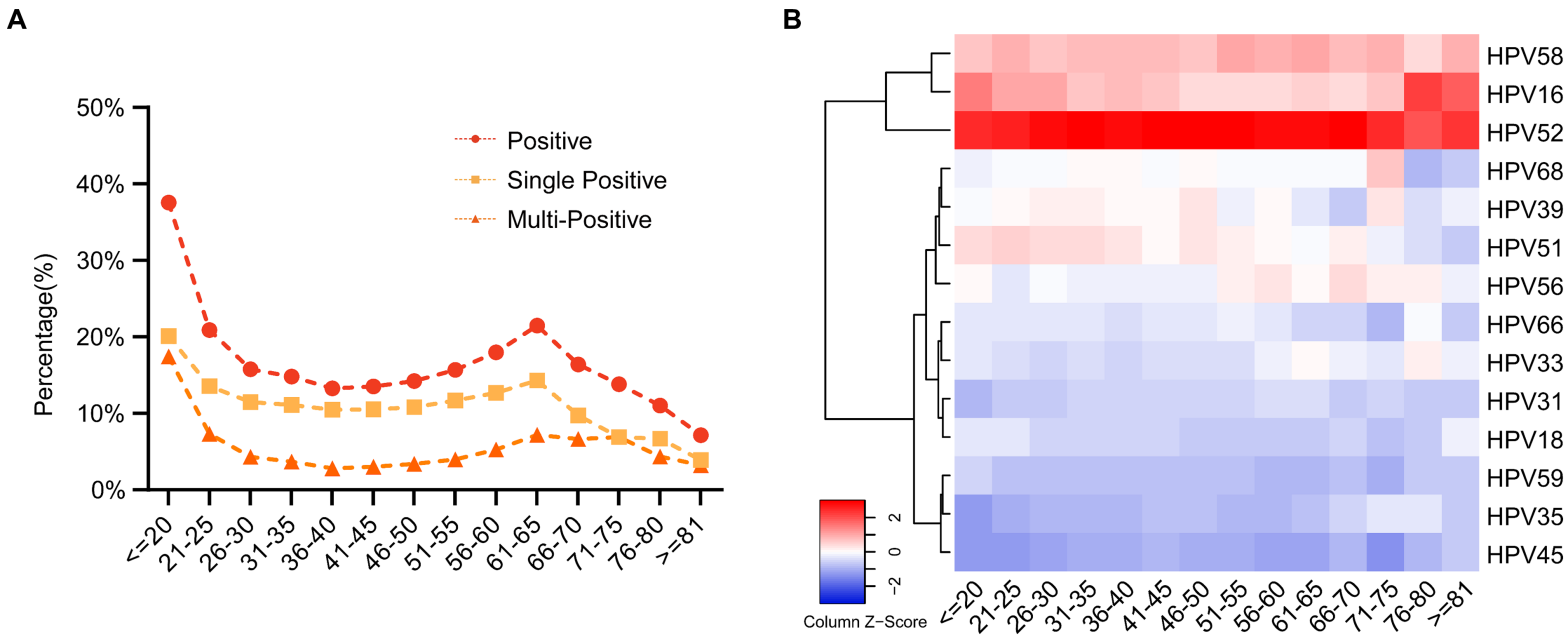
The prevalence of the HPV infection during 2016 and 2022. **(A)** The HPV prevalence and the ratio of single- and multiple infection over the 7 years. **(B)** The heatmap of HPV prevalence rates across different year groups and genotypes. Each row represented deviations of the point prevalence at each year from its own overall mean. The color reflected the magnitude of the deviation, Some HPV genotype prevalence decreased represented in blue, and others increased, represented in red. The darker the color, the larger difference from its overall mean.

Over the 7-year period from 2016 to 2022, the average prevalence of single positive HPV infections during these 7 years was 11.61% ± 1.04%, and the prevalence of multiple positive infections was 3.96% ± 0.5%. HPV experienced only a minor peak in 2018, despite the increasing absolute number of positive individuals over time (Figure 4A; Supplementary Table S5). In addition, we found there is no significant prevalence changes among different season (Supplementary Figure S1). We also observed that the prevalence of each genotype was quite stable over the 7-year period and the most changes can be observed for HPV68, HPV56 and HPV51 after 2018 (Figure 4B; Supplementary Table S5).

The COVID-19 pandemic emerged in late 2019, prompting China to swiftly adjust its epidemic prevention policies based on scientific assessments. These adjustments effectively protected the health of the population, facilitated economic recovery, and yielded significant outcomes. However, there is currently no available information regarding any disparities in HPV infections during the COVID-19 period compared to before the pandemic. To analyze this, we categorized the data from 2016 to 2019 as the “pre-COVID” period and the data from 2020 to 2022 as the “during-COVID” period. The results revealed a substantial disparity in HPV infections between these two periods (*p* = 0.000). Notably, we investigated the changes between HPV infections before and after the COVID-19 pandemic and specific HPV genotypes. Through stratified chi-square tests, we identified significant discrepancies in HPV infections during the pre- and during COVID periods for HPV genotypes 16, 18, 35, 45, 52, 58, 59, and 68 (Table 2).

**Table 2:** The HPV prevalence changes before and after COVID-19 pandemic.

HPV diagnosis	Pre-Covid	Post-Covid	Total	χ2	*p*.value
	2016–2019	2020–2022			
Negative	42,899 (38.91)	67,361 (61.09)	110,260	40.958	0.000
Positive	8,255 (41.31)	11,728 (58.69)	19,983		
Total	51,154 (39.28)	79,089 (60.72)	130,243		

## Discussion

Analyzing type-specific HPV prevalence and its distribution within specific regions is a crucial component in shaping effective prevention and control strategies aimed at reducing cervical cancer rates. While many previous studies have primarily focused on HPV infection within the broader female population, only a limited number have delved into the epidemiology of HPV infection in both males and females belonging to high-risk groups (11–14). Additionally, the impact of the COVID-19 pandemic on HPV prevalence has not been widely explored. In this study, we aim to address these questions and provide a comprehensive analysis of HPV infection trends. The overall prevalence of HPV positivity in this study was consistent with previous reports from Zhejiang (15) and other regions in China (16–18). The results also showed that the HPV positivity among the male subgroup was 11.51%, which was similar with the previous report (19, 20) but lower than that in other studies (21, 22). This difference may be due to the fact that the study population in the latter groups mainly came from sexually transmitted infection clinic. Our data suggest that HPV screening for males could be included in annual health examinations.

In this study, we showed that the most prevalent HPV genotypes in women was HPV52 (4.67%), followed by HPV58 (2.28%) and HPV16 (2.18%). The consistent results were found in all age groups. In addition, a study conducted from five randomly chosen counties in Zhejiang Province in 2010 showed that HPV-52 was the most prevalent type (3.1%), followed by HPV-16 (2.5%) and HPV58 (2.1%) (15). These discrepancies may be influenced by factors such as population growth, as observed in Hangzhou, where an increasing population has potentially contributed to changes in genotype prevalence. However, we found there was no significant prevalence changes among different season, which inconsistent with a previous study from the same province (11). Differences in sample size or source may have contributed to this inconsistency. Our findings also revealed that HPV infections exhibited significant regional variations in China, with HPV16, 52, and 58 being the most prevalent genotypes, albeit with notable differences across different cities. In some other regions of China, e.g., in Shanxi Province the most prevalent three genotypes were HPV16 (3%), HPV58 (1.9%) and HPV52 (0.8%) (17). In northern Guangdong Province, the most prevalent genotypes were HPV52 (4.16%), HPV16 (2.98%) and HPV58 (2.15%) (12). A survey of prevalence of HPV genotype in Yunnan Province showed that the three most prevalent genotypes were HPV52 (2.1%), HPV16 (1.7%) and HPV58 (1.0%) (23). A study from Northwest China showed that HPV 16 was most prevalent (5.18%), followed by HPV 58 (3.10%) and HPV 52 (2.75%) (24). The currently licensed HPV vaccines include bivalent vaccines (HPV16/18), quadrivalent vaccines (HPV16/18/6/11), and nine-valent vaccines (HPV16/18/6/11/31/33/45/52/58). Considering the most prevalent genotypes in China, both the bivalent and quadrivalent vaccine could not be effective in controlling HPV infection. Therefore, it is important to select a suitable vaccine (Table 3).

**Table 3:** The HPV genotype distribution changes before and after COVID-19 pandemic.

Genotype	Pre-Covid (2016–2019)		Post-Covid (2020–2022)		χ2	*p*.value	OR.value	OR.value 95% CI
	Negative (n)	Positive (n)	Negative (n)	Positive (n)				
HPV16	49,842	1,312	77,243	1846	6.987	0.01	0.908	0.845 ~ 0.975
HPV18	50,646	508	78,437	652	10.014	0.00	0.829	0.738 ~ 0.931
HPV31	50,721	433	78,417	672	0.004	0.98	1.004	0.889 ~ 1.133
HPV33	50,636	518	78,240	849	1.107	0.30	1.061	0.950 ~ 1.184
HPV35	50,872	282	78,744	345	8.584	0.00	0.79	0.675 ~ 0.925
HPV39	50,277	877	77,823	1,266	2.482	0.12	0.933	0.855 ~ 1.017
HPV45	51,003	151	78,910	179	5.828	0.02	0.766	0.617 ~ 0.952
HPV51	50,150	1,004	77,572	1,517	0.326	0.58	0.977	0.901 ~ 1.059
HPV52	48,420	2,734	75,214	3,875	12.776	0.00	0.912	0.868 ~ 0.959
HPV56	50,445	709	77,903	1,186	2.794	0.10	1.083	0.986 ~ 1.190
HPV58	49,723	1,431	77,229	1860	25.048	0.00	0.837	0.780 ~ 0.897
HPV59	50,787	367	78,685	404	22.536	0.00	0.711	0.617 ~ 0.819
HPV66	50,539	615	78,194	895	1.352	0.25	0.941	0.848 ~ 1.043
HPV68	50,523	631	77,712	1,377	52.715	0.00	1.419	1.290 ~ 1.560

Age emerged as a critical factor in HPV infection prevalence, aligning with previous research (11, 12, 25). High-risk HPV infection rates tended to be higher among individuals below 20 and those above 61 years old. These variations are multifaceted, with younger individuals potentially at greater risk due to sexual behavior, while older individuals face challenges in clearing HPV due to physiological and immunological changes. The prevention strategies, including vaccination for young individuals and potential adjustments in cervical cancer screening age limits, are essential to effectively combat HPV-related health risks.

This study encompassed patients from both health examination centers and gynecology clinics, providing a more representative reflection of the overall HPV infection situation. We observed an increase in the overall infection rate during the first 3 years, followed by a slight decrease, despite an increase in the total number of positive cases. During the COVID-19 pandemic, healthcare systems in many regions were overwhelmed with COVID-19 cases. As a result, non-urgent medical services, including routine screenings such as HPV tests and cervical cancer screenings, were postponed or canceled. This interruption in healthcare services could lead to delays in detecting and managing HPV infections and related conditions, potentially affecting the prevalence of HPV. In addition, The COVID-19 pandemic led to changes in social behavior, including lockdowns, social distancing measures, and changes in sexual behavior. These factors could have indirect effects on the transmission of HPV. For example, reduced social interactions may lead to fewer sexual encounters, potentially reducing the spread of the virus.

However, our study has limitations. It is a cross-sectional study with limited access to cervical pathological diagnoses and follow-up data. The sample size for males was relatively small due to awareness issues surrounding HPV screening in this population. In addition, our dataset lack of morphological results and correlation with HPV genotypes as described previously (26). Furthermore, the reasons behind the significant changes in HPV prevalence and distribution pre- and during COVID-19 pandemic are complex and warrant further investigation.

In conclusion, our comprehensive analysis of HPV infection prevalence and genotype distribution in a large study population has yielded several noteworthy findings. We observed a relatively high prevalence of HPV infection, with 15.34% of the overall study population affected. The distribution of high-risk genotypes was consistent between male and female groups, with HPV16, HPV52, HPV58, HPV18, and HPV39 being among the most commonly identified genotypes. Age-related trends in HPV prevalence unveiled a bimodal pattern, and the impact of the COVID-19 pandemic on HPV epidemiology was evident. Our study contributes valuable insights to our understanding of HPV epidemiology and underscores the importance of ongoing surveillance and preventive measures to reduce the burden of HPV-related diseases. Further research is needed to explore the complex factors driving these trends.

## Data availability statement

The raw data supporting the conclusions of this article will be made available by the authors, without undue reservation.

## Ethics statement

The studies involving humans were approved by the present study IRB was approved by the First People’s Hospital of Linping District. The studies were conducted in accordance with the local legislation and institutional requirements. Written informed consent for participation in this study was provided by the participants’ legal guardians/next of kin.

## Author contributions

JW: Investigation, Methodology, Writing – original draft, Writing – review & editing, Data curation, Formal analysis, Resources. KZ: Data curation, Formal analysis, Investigation, Methodology, Writing – original draft, Writing – review & editing, Software, Validation, Visualization. JX: Data curation, Writing – original draft, Writing – review & editing, Project administration. FH: Data curation, Project administration, Writing – original draft, Writing – review & editing. NC: Data curation, Project administration, Writing – original draft, Writing – review & editing. WW: Data curation, Writing – original draft, Writing – review & editing, Formal analysis, Visualization. YM: Writing – original draft, Writing – review & editing, Conceptualization, Funding acquisition, Investigation, Methodology, Resources, Supervision. XS: Conceptualization, Funding acquisition, Investigation, Methodology, Supervision, Writing – original draft, Writing – review & editing, Validation.

## Footnotes

^1^http://heatmapper.ca/
