# Supplementary material for: Prevalence and genotype distribution of HPV infection from Hangzhou of Zhejiang Province pre- and during COVID-19 pandemic
Source: Front Public Health. 2024 May 30;12:1357311. doi: 10.3389/fpubh.2024.1357311 (PMC11169856; doi:10.3389/fpubh.2024.1357311)
Supplement: Supplementary file 1 [file Data_Sheet_1.docx]

**Supplemental Table 1. Comparison of the HPV genotype distribution between male and female individuals.**

| **Genotype** | **Gender** | **Male** | | **Female** | | **χ2** | ***p .value*** | **OR  .value** | **OR .value 95% CI** |
| --- | --- | --- | --- | --- | --- | --- | --- | --- | --- |
|  |  | **Negative(n)** | **Positive(n)** | **Negative(n)** | **Positive(n)** |  |  |  |  |
| HPV16 | | 581 | 10 | 126504 | 3148 | 1.347 | 0.284 | 1.446 | 0.773 ~ 2.704 |
| HPV18 | | 585 | 6 | 128498 | 1154 | 0.104 | 0.659 | 0.876 | 0.391 ~ 1.961 |
| HPV31 | | 588 | 3 | 128550 | 1102 | 0.820 | 0.500 | 1.680 | 0.540 ~ 5.233 |
| HPV33 | | 584 | 7 | 128292 | 1360 | 0.104 | 0.683 | 0.884 | 0.419 ~ 1.867 |
| HPV35 | | 590 | 1 | 129026 | 626 | 1.208 | 0.540 | 2.863 | 0.402 ~ 20.387 |
| HPV39 | | 582 | 9 | 127518 | 2134 | 0.055 | 1.000 | 1.082 | 0.559 ~ 2.093 |
| HPV45 | | 590 | 1 | 129323 | 329 | 0.166 | 1.000 | 1.501 | 0.210 ~ 10.705 |
| HPV51 | | 584 | 7 | 127138 | 2514 | 1.765 | 0.229 | 1.650 | 0.782 ~ 3.479 |
| HPV52 | | 569 | 22 | 123065 | 6587 | 2.252 | 0.158 | 1.384 | 0.904 ~ 2.121 |
| HPV56 | | 582 | 9 | 127766 | 1886 | 0.019 | 0.862 | 0.955 | 0.493 ~ 1.847 |
| HPV58 | | 577 | 14 | 126375 | 3277 | 0.060 | 1.000 | 1.069 | 0.628 ~ 1.818 |
| HPV59 | | 588 | 3 | 128884 | 768 | 0.072 | 1.000 | 1.168 | 0.375 ~ 3.640 |
| HPV66 | | 584 | 7 | 128149 | 1503 | 0.003 | 0.847 | 0.978 | 0.464 ~ 2.065 |
| HPV68 | | 582 | 9 | 127653 | 1999 | 0.001 | 1.000 | 1.013 | 0.523 ~ 1.959 |

**Supplemental Table 2.** **Association of HPV genotype with clinical diagnosis “menstrual disorders” in female individuals.**

| **Genotype** | **Clinical diagnosis** | **Non-Menstrual disorders** | | **Menstrual disorders** | | **χ2** | ***p .value*** | **OR  .value** | **OR .value 95% CI** |
| --- | --- | --- | --- | --- | --- | --- | --- | --- | --- |
|  | **HPV diagnosis** | **Negative(n)** | **Positive(n)** | **Negative(n)** | **Positive(n)** |  |  |  |  |
| HPV16 | | 119086 | 2995 | 7418 | 153 | 5.627 | 0.017 | 0.820 | 0.696 ~ 0.966 |
| HPV18 | | 120984 | 1096 | 7514 | 58 | 1.404 | 0.256 | 0.852 | 0.654 ~ 1.111 |
| HPV31 | | 121037 | 1043 | 7513 | 59 | 0.478 | 0.561 | 0.911 | 0.700 ~ 1.186 |
| HPV33 | | 120783 | 1297 | 7509 | 63 | 3.647 | 0.055 | 0.781 | 0.606 ~ 1.007 |
| HPV35 | | 121492 | 588 | 7534 | 38 | 0.061 | 0.797 | 1.042 | 0.750 ~ 1.448 |
| HPV39 | | 120075 | 2005 | 7443 | 129 | 0.165 | 0.675 | 1.038 | 0.867 ~ 1.242 |
| HPV45 | | 121766 | 314 | 7557 | 15 | 0.984 | 0.408 | 0.770 | 0.458 ~ 1.293 |
| HPV51 | | 119716 | 2364 | 7422 | 150 | 0.074 | 0.764 | 1.023 | 0.866 ~ 1.209 |
| HPV52 | | 115850 | 6230 | 7215 | 357 | 2.231 | 0.138 | 0.92 | 0.825 ~ 1.026 |
| HPV56 | | 120303 | 1777 | 7463 | 109 | 0.013 | 0.961 | 0.989 | 0.814 ~ 1.201 |
| HPV58 | | 118964 | 3116 | 7411 | 161 | 5.256 | 0.021 | 0.829 | 0.707 ~ 0.973 |
| HPV59 | | 121343 | 737 | 7541 | 31 | 4.571 | 0.031 | 0.677 | 0.472 ~ 0.970 |
| HPV66 | | 120649 | 1431 | 7500 | 72 | 3.048 | 0.086 | 0.809 | 0.638 ~ 1.027 |
| HPV68 | | 120178 | 1902 | 7475 | 97 | 3.603 | 0.061 | 0.820 | 0.668 ~ 1.007 |

**Supplemental Table 3. Association of HPV genotype with clinical diagnosis “reproductive inflammation” in female individuals.**

| **Genotype** | **Clinical diagnosis** | **Non-Reproductive system inflammation** | | **Reproductive system inflammation*** | | **χ2** | ***p .value*** | **OR  .value** | **OR .value 95% CI** |
| --- | --- | --- | --- | --- | --- | --- | --- | --- | --- |
|  | **HPV diagnosis** | **Negative(n)** | **Positive(n)** | **Negative(n)** | **Positive(n)** |  |  |  |  |
| HPV16 | | 100104 | 2432 | 26981 | 726 | 5.689 | 0.018 | 1.108 | 1.018 ~ 1.205 |
| HPV18 | | 101668 | 868 | 27415 | 292 | 10.625 | 0.001 | 1.248 | 1.092 ~ 1.425 |
| HPV31 | | 101684 | 852 | 27454 | 253 | 1.752 | 0.184 | 1.100 | 0.955 ~ 1.266 |
| HPV33 | | 101466 | 1070 | 27410 | 297 | 0.169 | 0.690 | 1.028 | 0.903 ~ 1.169 |
| HPV35 | | 102070 | 466 | 27546 | 161 | 7.298 | 0.008 | 1.280 | 1.070 ~ 1.532 |
| HPV39 | | 100878 | 1658 | 27222 | 485 | 2.401 | 0.123 | 1.084 | 0.979 ~ 1.200 |
| HPV45 | | 102294 | 242 | 27619 | 88 | 5.746 | 0.018 | 1.347 | 1.055 ~ 1.720 |
| HPV51 | | 100576 | 1960 | 27146 | 561 | 1.473 | 0.229 | 1.060 | 0.965 ~ 1.166 |
| HPV52 | | 97353 | 5183 | 26281 | 1426 | 0.382 | 0.537 | 1.019 | 0.960 ~ 1.082 |
| HPV56 | | 101088 | 1448 | 27260 | 447 | 6.154 | 0.014 | 1.145 | 1.029 ~ 1.274 |
| HPV58 | | 99996 | 2540 | 26956 | 751 | 4.822 | 0.029 | 1.097 | 1.010 ~ 1.191 |
| HPV59 | | 101945 | 591 | 27527 | 180 | 1.990 | 0.158 | 1.128 | 0.954 ~ 1.333 |
| HPV66 | | 101381 | 1155 | 27352 | 355 | 4.563 | 0.034 | 1.139 | 1.011 ~ 1.284 |
| HPV68 | | 101020 | 1516 | 27215 | 492 | 12.694 | 0.000 | 1.205 | 1.087 ~ 1.335 |

*** Reproductive system inflammations include vaginitis, cervicitis, vulvitis, pelvic inflammatory disease (PID), salpingitis, and balanitis.**

|  | **<=20** | **21-25** | | **26-30** | | **31-35** | | **36-40** | | **41-45** | | **46-50** | | **51-55** | **56-60** | **61-65** | **66-70** | **71-75** | | **76-80** | | **>=81** | |
| --- | --- | --- | --- | --- | --- | --- | --- | --- | --- | --- | --- | --- | --- | --- | --- | --- | --- | --- | --- | --- | --- | --- | --- |
| Group Number | 940 | 7643 | 20697 | | 27501 | | 20370 | | 17281 | | 14821 | | 9937 | | 5464 | 2832 | 1550 | 754 | 299 | | 154 | |  |
| HPV Positive | 353(37.55) | 1597(20.89) | | 3263(15.77) | | 4068(14.79) | | 2704(13.27) | | 2338(13.53) | | 2108(14.22) | | 1559(15.69) | 983(17.99) | 608(21.47) | 254(16.39) | 104(13.79) | | 33(11.04) | | 11(7.14) | |
| HPV Single Positive | 189(20.11) | 1037(13.57) | | 2373(11.47) | | 3052(11.10) | | 2135(10.48) | | 1816(10.51) | | 1602(10.81) | | 1162(11.69) | 694(12.70) | 405(14.30) | 151(9.74) | 52(6.90) | | 20(6.69) | | 6(3.90) | |
| HPV Multi Positive | 164(17.45) | 560(7.33) | | 890(4.30) | | 1016(3.69) | | 569(2.79) | | 522(3.02) | | 506(3.41) | | 397(4.00) | 289(5.29) | 203(7.17) | 103(6.65) | 52(6.90) | | 13(4.35) | | 5(3.25) | |
| HPV16 | 96(3.04) | 309(9.78) | | 595(18.84) | | 602(19.06) | | 409(12.95) | | 335(10.61) | | 282(8.93) | | 211(6.68) | 136(4.31) | 101(3.20) | 42(1.33) | 21(0.66) | | 14(0.44) | | 5(0.16) | |
| HPV18 | 39(3.36) | 141(12.16) | | 198(17.07) | | 232(20.00) | | 147(12.67) | | 139(11.98) | | 97(8.36) | | 75(6.47) | 43(3.71) | 27(2.33) | 14(1.21) | 6(0.52) | | 1(0.09) | | 1(0.09) | |
| HPV31 | 20(1.81) | 97(8.78) | | 158(14.30) | | 236(21.36) | | 140(12.67) | | 121(10.95) | | 114(10.32) | | 85(7.69) | 72(6.52) | 41(3.71) | 12(1.09) | 8(0.72) | | 1(0.09) | | 0(0.00) | |
| HPV33 | 38(2.78) | 123(9.00) | | 200(14.63) | | 260(19.02) | | 145(10.61) | | 149(10.90) | | 148(10.83) | | 109(7.97) | 85(6.22) | 68(4.97) | 25(1.83) | 11(0.80) | | 5(0.37) | | 1(0.07) | |
| HPV35 | 10(1.59) | 47(7.50) | | 91(14.51) | | 112(17.86) | | 84(13.40) | | 89(14.19) | | 71(11.32) | | 44(7.02) | 28(4.47) | 24(3.83) | 13(2.07) | 11(1.75) | | 3(0.48) | | 0(0.00) | |
| HPV39 | 45(2.10) | 188(8.77) | | 375(17.50) | | 447(20.86) | | 279(13.02) | | 229(10.69) | | 252(11.76) | | 139(6.49) | 105(4.90) | 52(2.43) | 12(0.56) | 17(0.79) | | 2(0.09) | | 1(0.05) | |
| HPV45 | 10(3.03) | 28(8.48) | | 46(13.94) | | 67(20.30) | | 44(13.33) | | 58(17.58) | | 32(9.70) | | 30(9.09) | 7(2.12) | 3(0.91) | 5(1.52) | 0(0.00) | | 0(0.00) | | 0(0.00) | |
| HPV51 | 62(2.46) | 246(9.76) | | 447(17.73) | | 538(21.34) | | 323(12.81) | | 247(9.80) | | 250(9.92) | | 185(7.34) | 108(4.28) | 66(2.62) | 35(1.39) | 12(0.48) | | 2(0.08) | | 0(0.00) | |
| HPV52 | 126(1.91) | 508(7.69) | | 1067(16.14) | | 1330(20.12) | | 841(12.73) | | 763(11.54) | | 701(10.61) | | 525(7.94) | 345(5.22) | 232(3.51) | 113(1.71) | 39(0.59) | | 13(0.20) | | 6(0.09) | |
| HPV56 | 50(2.64) | 150(7.92) | | 306(16.15) | | 345(18.21) | | 215(11.35) | | 203(10.71) | | 184(9.71) | | 175(9.23) | 129(6.81) | 73(3.85) | 43(2.27) | 16(0.84) | | 5(0.26) | | 1(0.05) | |
| HPV58 | 71(2.16) | 295(8.96) | | 496(15.07) | | 657(19.96) | | 415(12.61) | | 365(11.09) | | 320(9.72) | | 284(8.63) | 179(5.44) | 127(3.86) | 50(1.52) | 23(0.70) | | 6(0.18) | | 3(0.09) | |
| HPV59 | 32(4.15) | 87(11.28) | | 135(17.51) | | 138(17.90) | | 102(13.23) | | 76(9.86) | | 82(10.64) | | 64(8.30) | 28(3.63) | 15(1.95) | 8(1.04) | 3(0.39) | | 1(0.13) | | 0(0.00) | |
| HPV66 | 39(2.58) | 137(9.07) | | 248(16.42) | | 299(19.80) | | 174(11.52) | | 169(11.19) | | 167(11.06) | | 133(8.81) | 82(5.43) | 38(2.52) | 15(0.99) | 5(0.33) | | 4(0.26) | | 0(0.00) | |
| HPV68 | 41(2.04) | 175(8.72) | | 310(15.44) | | 406(20.22) | | 277(13.79) | | 219(10.91) | | 221(11.01) | | 152(7.57) | 97(4.83) | 61(3.04) | 28(1.39) | 21(1.05) | | 0(0.00) | | 0(0.00) | |

Supplemental Table 4 The prevalence and genotype distribution of HPV in different age groups

Supplemental Table 5 The prevalence and genotype distribution of HPV in different years

| Years | 2016(N=8870) | 2017(N=10360) | 2018(N=12525) | 2019(N=19399) | 2020(N=21852) | 2021(N=27034) | 2022(N=30203) |
| --- | --- | --- | --- | --- | --- | --- | --- |
| HPV Positive | 1327(14.96) | 1672(16.14) | 2182(17.42) | 3074(15.85) | 3356(15.36) | 4116(15.23) | 4256(14.09) |
| HPV Single Positive | 1043(11.76) | 1319(12.73) | 1648(13.16) | 2168(11.18) | 2453(11.23) | 3011(11.14) | 3052(10.10) |
| HPV Multi Positive | 284(3.20) | 353(3.41) | 534(4.26) | 906(4.67) | 903(4.13) | 1105(4.09) | 1204(3.99) |
| HPV16 | 221(2.49) | 255(2.46) | 330(2.63) | 506(2.61) | 564(2.58) | 671(2.48) | 611(2.02) |
| HPV18 | 105(1.18) | 132(1.27) | 126(1.01) | 145(0.75) | 175(0.8) | 224(0.83) | 253(0.84) |
| HPV31 | 62(0.7) | 89(0.86) | 114(0.91) | 168(0.87) | 189(0.86) | 213(0.79) | 270(0.89) |
| HPV33 | 54(0.61) | 70(0.68) | 109(0.87) | 285(1.47) | 243(1.11) | 287(1.06) | 319(1.06) |
| HPV35 | 47(0.53) | 63(0.61) | 88(0.7) | 84(0.43) | 102(0.47) | 112(0.41) | 131(0.43) |
| HPV39 | 135(1.52) | 218(2.1) | 215(1.72) | 309(1.59) | 349(1.6) | 438(1.62) | 479(1.59) |
| HPV45 | 28(0.32) | 22(0.21) | 46(0.37) | 55(0.28) | 53(0.24) | 61(0.23) | 65(0.22) |
| HPV51 | 167(1.88) | 205(1.98) | 261(2.08) | 371(1.91) | 407(1.86) | 526(1.95) | 584(1.93) |
| HPV52 | 389(4.39) | 547(5.28) | 718(5.73) | 1080(5.57) | 1125(5.15) | 1359(5.03) | 1391(4.61) |
| HPV56 | 103(1.16) | 112(1.08) | 158(1.26) | 336(1.73) | 357(1.63) | 419(1.55) | 410(1.36) |
| HPV58 | 253(2.85) | 270(2.61) | 435(3.47) | 473(2.44) | 526(2.41) | 648(2.4) | 686(2.27) |
| HPV59 | 71(0.8) | 99(0.96) | 108(0.86) | 89(0.46) | 109(0.5) | 127(0.47) | 168(0.56) |
| HPV66 | 95(1.07) | 133(1.28) | 148(1.18) | 239(1.23) | 263(1.2) | 321(1.19) | 311(1.03) |
| HPV68 | 58(0.65) | 51(0.49) | 146(1.17) | 376(1.94) | 387(1.77) | 502(1.86) | 488(1.62) |
