# Supplementary figures and images for: Prevalence and genotype distribution of HPV infection from Hangzhou of Zhejiang Province pre- and during COVID-19 pandemic
Source: Front Public Health. 2024 May 30;12:1357311. doi: 10.3389/fpubh.2024.1357311 (PMC11169856; doi:10.3389/fpubh.2024.1357311)

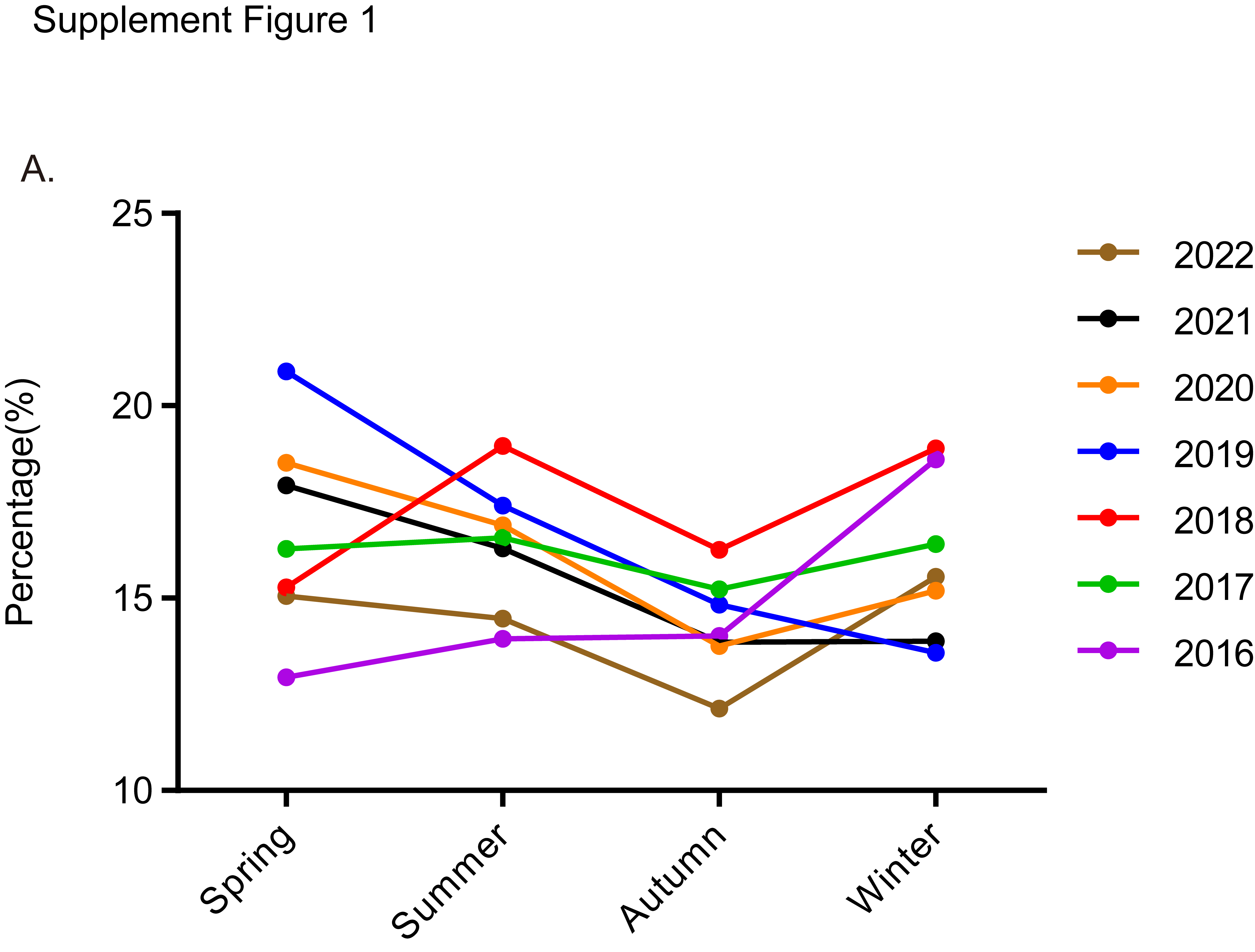

Supplement: Supplementary file 2 [file Image_1.TIF]
